# Oral health and socio-economic status among children during Syrian crisis: a cross-sectional study

**DOI:** 10.1186/s12903-019-0856-8

**Published:** 2019-07-25

**Authors:** Bahaa Aldin Alhaffar, Raeed Alawabdi, Leen Barakat, Chaza Kouchaji

**Affiliations:** 1 Faculty of Dentistry, Department of Periodontology, Damascus University, Alkhateeb sq, Damascus, Syria; 2 Faculty of Dentistry, Department of Cosmetic Dentistry, Damascus University, Damascus, Syria; 3 Faculty of Dentistry, Damascus University, Damascus, Syria; 4 Faculty of Dentistry, Pediatric Dentistry Department, Damascus University, Damascus, Syria

**Keywords:** Oral health, DMFT index, Socioeconomic status, Syrian crisis

## Abstract

**Background:**

The Syrian crisis has started eight years ago and has, directly and indirectly, affected all the aspects of the Syrians lives. A lot of new war-related factors contributed to change the socio-economic status, the demographical distribution and the ability to access the public health services. Moreover, the crisis created the biggest displacement crisis both inside and outside Syria. Therefore, it is important to study the prevalence of dental caries and oral health in these specific circumstances in order to build a database to assess and compare future results of preventive programs and to assess health and social needs of the communities affected by war or crisis. The aim of this research is to Study the level of oral health among children during the Syrian crisis, as well as the relation between oral health and socioeconomic status (SES).

**Methods:**

A cross-sectional study to assess the oral health of children in Damascus city by using DMFT index and other dental indices. The data were collected from ten randomly selected schools covering all the areas of Damascus city, and the final sample size was 811 children.

**Results:**

DMFT index was used to assess the oral health of the children. The average number was (3.36) among all children; 14% of the sample size had a good oral health, while 86% had at least one decayed, missed, or filled tooth. There was also a strong association between SES of the child and the oral health represented as DMFT Index (*P* = 0.03), Pearson’s correlation test displayed an inverse association between the SES and oral health (*P* = − 0.074).

**Conclusion:**

This study highlights the impact of the Syrian crisis on the SES of the Syrian children and their oral health. Bad oral health has been recorded and it has a significant relation with the SES of the children.

## Background

The Syrian crisis began in 2011 and has had serious consequences on Syrians. There are roughly 13 million people currently in need of medical services, and the requirements necessary to helping these people have exceeded the available resources. WHO (World Health Organization) has only received 1/3 of the funding needed to implement the Humanitarian Activities Plan for 2016 [1]. Moreover, as a result of the crisis, many healthcare providers were reported as killed, fled out of the country, or have lost their workplace [2]. The decrease in health and education services have been devastating to the country and its people [3]. Syrian citizens are facing extremely difficult economic conditions, which affecting their capacity to survive by themselves and provide the needed support for their families [4].

Despite the importance of oral health and its effects on general health, oral health has been completely neglected during the Syrian crisis. The relative cost and complexity of dental health services made it very hard for people to access oral health [5]. A research published in 2019 has reported a higher rate of dental caries than expected and the prevalence among children was 79.1% with an average DMFT of (2.03) [6].

Caries, are the most common chronic disease of oral health diseases, and according to WHO they are the main cause of tooth pain and loss for children [7]. Decays are located on the surface of the tooth and It has a clear pathogenesis, the microorganisms cause the tooth minerals to decay, and if left untreated may lead to tooth loss [8]. Dental decays start as a white spot of demineralization and the progression of the white spot to dental decays is determined upon the balance between demineralization and remineralization [9].

Decays are widespread throughout the world; it is a general health problem in several countries [10]. Studies have shown that poor oral health also leads to different systemic diseases such as cardiovascular and respiratory diseases [11, 12]. The prevalence of tooth decays differs among countries, as it is more prevalent in less developed countries due to poorer socioeconomic status [13], as well as environmental factors [14]. Decays are complex and socioeconomic status is an important risk factor for the occurrence of decays especially in children [15].

There is a direct association between education level and oral health in children. Higher education levels provide a better understanding of oral health and general health [16]. Individuals with a low level of education tend to have a higher prevalence of decays than their counterparts [17], and so as children whose mothers had a high educational level tend to have a better oral health than the children whose mothers had a lower level [18].

Therefore, it is important to study the prevalence of tooth decays and oral health as an indicator for the general health, especially during this period of time in Syria.

### Aims of the research

- Studying the level of oral health among children during the Syrian crisis.
- Studying the implications of the socio-economic status on oral health.

## Methods

### Research methodology

A cross-sectional research was carried out to study the prevalence of caries among children and its relation to (SES).

### Sampling

The original sample population includes seventh-grade schoolchildren, most of the registrants are (12) years old. This age is considered very important as it is the age when the child moves from primary school; which means they this is the last age in which a reliable sample can be collected easily through the school system. It is also more likely that most of the permanent teeth (except the third molar) have emerged. Therefore, this age group was chosen to express significant global comparisons and orientations of diseases [19]. The sample size was calculated based on the recorded numbers in the Directorate of Education in Damascus. Simple random sampling technique was used to select the schools from which the sample would be collected to cover all areas of the city. Damascus has a total of (1200) schools, about (145) of them include seventh grade classes. The number of students enrolled in the seventh grade during the academic year (2017–2018) was about (35 thousand) students [20]. The final sample size was (811) children. The median age was (12.5) The number of males was 431 (53.1%) and females 380 (46.9%). With a 95% confidence level and a (3.39±) confidence interval.

### School selection criteria

The schools were selected according to the geographical distribution of the areas in Damascus city. The capital was divided into five areas (Northeast, Northwest, South-East, South-West and Downtown) and Ten schools were randomly selected from the final list which was provided by the ministry of education, this official list contains only the schools which have both the residents students in the area and new students who lost their houses from around Damascus, in order to represent the general population in the sample size, the random selection was made using a simple Excel equation to select two schools from each area in the city.

Moreover, all parts of Damascus city have a similar access to public health services and to governmental hospitals. The government has distributed the internal displaced people across all the parts of Damascus in almost equal numbers and the refuges were mostly residents in temporary residential places created by the government around Damascus. Finally, all parts of the city have the same socioeconomic status and have been affected with the same circumstances during the Syrian crisis.

### Student selection criteria

Each school had a different number of seven-grade classes. Two seven-grade classes were randomly selected, and all students who signed consent form have been examined to collect the oral indices. Because of the war, not all students were of the same age, some were older or younger than the age of the seventh grade, which is normally 12 years old. The reason behind this is directly related to the Ministry of Education’s merging process, that aims to give students a chance to continue their education after dropping out schools because of the crisis in Syria.

There was no difference between the displaced students who lost their houses and moved to other areas, and the students who are residents in the host community where the school is located the research question might be sensitive to the students during the time of the data collection.

### Research procedure

Consent papers have been given to the students on the first day in order to obtain their parents approvals, and data has been collected from the children whom their parents agreed to participate in this study on the second day. All children who didn’t show the approval paper have not been included in the study. The socio-economic status questionnaire was distributed and the students’ oral examination was done according to WHO guidelines for conducting surveys, which was published in the Oral Health Surveys Basic Methods fifth edition. Data were collected during the period from 11 September to 19 November 2017. All the dentist participated in this study were from Damascus University; seven dentists in total collected the data. One dentist has a master degree in oral medicine and he was responsible for the oral examination which means that all the examinations were carried out by one dentist. Two dentists have a master degree in periodontology and they were responsible for the oral hygiene instructions and to teach the children how to brush their teeth. Moreover, four dentists were fifth-year students during the time of the data collection and they helped to collect the data and handled other organizational duties during the data collection in schools.

The oral examination was done in each school for all the students with signed consent paper, a separated room in the school was allocated and all protective and infection control procedure were taken in consideration.

#### Materials used

A special data collection questionnaire is used, and it consist of three sections:

1. Personal Information (Demographic Data): Includes gender, age, current and previous residence, and current grade.
2. Assessment of the Socio-economic status: The evaluation of the socioeconomic status was assessed by five questions. The questions were related to first, − the education of both the father and the mother of the children, second, the monthly family income, and third, profession of father and mother. Based on the previous questions, the SES was divided into 3 categories (high, moderate and low) [21, 22].
3. The number of missing, decayed and filled teeth in children according to DMFT index, gingival index according to Löe-Silence, and plaque and periodontitis index according to Ramfjord.

#### Examination tools

Gingival probe (WHO probe), disposable dental explorer, disposable dental mirror, Cotton rollers, Personal protective equipment (PPE).

### Ethical approvals

Ethical approval has been obtained from the Ministry of Higher Education, the School Health Directorate, and the Deanship of the Faculty of Dentistry.

### Statistical analysis

The data was analyzed using SPSS V.22, using descriptive statistics (percentages, means, median, standard deviations) in addition to using inferential statistics using (T-test, ANOVA, Pearson’s correlation).

## Results

Data analysis showed a high rate of DMFT among children in Damascus city, the average DMFT was (3.36) and it ranged between (0–13). Fourteen percent of the included sample had good oral health and did not show any decayed, missed, or filled teeth, while the rest (86%) of the sample had at least on oral health problem.

The average number of decayed teeth was (2.93),it ranged between (0–12) and the caries prevalence in the city was (83%). Females had a slightly higher rate of the average DMFT value (3.56) compared to males (3.19) (Table 1).

**Table 1:** The average value of the DMFT index according to the gender

The average value	Children who have a decayed, missed or filled teeth		Children who do not have a decayed, missed or filled teeth		variables
	%	the number	%	the number	
3.19	446%.	362	85%.	69	**male**
3.56	414%.	335	55%.	45	**female**
–	86%	697	14%	114	**Total**

However, T-test didn’t show a significant difference between the two variables (*P* = 0.075). Moreover, ANOVA test did not show any significant difference between the oral health represented as DMFT average value and the geographical distribution of the data (*P* = 0.063), which means there is no difference between the sectors of the city in the matter of oral health (Table 2).

**Table 2:** Statistical tests to study the relationship between SES and the level of oral health

Variable		Average DMFT	Percentage of the sample	Statistical test	*P* value
gender	male	3.19	53.1%	T-test	0.075
	female	3.56	46.9%		
Socio-economic status	Low	5.65	44.1%	ANOVA Test	0.03
	Moderate	3.85	39.6%		
	High	2.43	16.3%	Pearson’s correlation	- 0.074
Geographical area	Northeast	4.03	21.8%	ANOVA	0.063
	Northwest	3.5	19.5%		
	South-East	3.86	21.2%		
	South- west	2.89	22.5%		
	Downtown	2.53	15.6%		

The average number of missed teeth was 0.48 and the average number of filled teeth was 0.02. The average plaque index value was 0.79 and the gingival index value was 0.85.

According to the level of SES the children divided into three levels, first, children with low SES (44.1%), second, children with moderate SES (39.6%), and third children with high SES (16.3%).

**Fig. 1:**
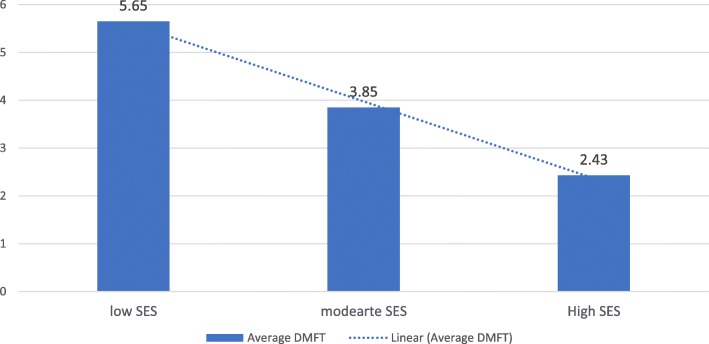
Relation between (SES) and Average DMFT

ANOVA test showed a significant association between oral health and the level of (SES) was found (*P* = 0.03), and the average DMFT for the low SES was (5.65), moderate SES (3.85), and for the high SES level (2.43). The correlation between the two variables according to Pearson’s correlation is significant and negative *p* = − 0.074, the lower the SES gets, the value of DMFT will be increased, which indicates poor oral health. Figure 1 shows the relationship between DMFT index and SES.

## Discussion

Studies over the past decade have shown a remarkable difference in oral health among individuals with higher social and economic status compared to individuals with lower social and economic status [19]. In this study, the assessment of the Socio-economic status was using the family income, father and mother level of education, and profession of both the father and mother, and in this study, almost half of the sample have a low socio-economic status. While other used many other indicators to assess the social and economic status, one of the most important indicators that can be used is occupation, family income and level of education. Each indicator covers a different aspect of social classes [20]. Income and level of education is a tool frequently used to measure social and economic status, as they are primarily responsible for securing the basic necessities of health. The current income is shown through a direct appreciation of the conditions of life, and health habits and behavior, which also reflects the individual’s position and style in society [23].

The difference in income is closely related to mortality and disease incidence, including oral diseases, the low rate of both income and year of the study resulted in an increased in the risk of oral diseases [17]. Previous studies conducted on a number of adults showed that member of the lower class have lower oral health compared to those in the higher class who tend to take constant care of their health [24].

In addition, in a study of the economic and social context of the spread of the need for dental treatment, the need for higher-income individuals to treat was less [25]. And in this research, the result was similar to the previous study, and we found that lower overall oral health can be found in children from low SES.

The low economic and social situation constituted about 50% of the factors influencing the prevalence of tooth caries and gingivitis at 12 years old. Which is closely linked to health, behavioral change and lifestyle, so they are emphasized in preventive methods [15].

in this research the average DMFT was 3.36 which is considered higher prevalence compared to the WHO recommendations. The prevalence of dental caries among children in low social and economic status schools is associated with oral health behavior and eating habits. The social and economic situation is a high-risk factor for children’s teeth [26].

Although the role of parents regarding the oral health and the low level of social services was not statistically significant, there was an indirect relationship between the role of parents in oral health education and eating habits [27].

In a study conducted in India on 12-year-old children, the prevalence and severity of dental caries were low among children in urban areas compared to the less developed. The percentage of boys was greater than that of girls. It was observed that there was no methodology and regularity in oral care procedures and dental clinics are not visited frequently. The presence of dental caries is markedly visible as it is linked to gender, place of residence, frequency of use of brush, frequency of consumption of carbonated beverages and sweets, parenting and education [28].

There is great concern today about the dental health of poorer children deprived of health services as recent data show that these children are more likely to be exposed to dental caries. For example, studies have shown that children living in poor and non-fluorinated communities in the United Kingdom have a lot of caries compared to those who live in better conditions within the fluorinated communities [29].

A study in Korea in 2008–2010 showed that the number of dental brushing varied depending on socio-economic factors and noted that the persons involved in the research had a higher income and were more interested in brushing their teeth. It also explained that higher-income participants and high-level educational recipients were more interested in using secondary oral care products such as dental floss and intraoral brushes, mouth rinses (chlorhexidine) and electric brushes [30].

WHO has determined that the number of dental caries must not exceed three in children at 12 years, and we have found in our study a value higher than the global value [31]. The previous national survey showed a significant prevalence of caries and poor oral health, with only 6–15% of the individuals safe from oral diseases such as caries and gingivitis [32].

A large proportion of the 15-year-olds had a high concentration of calculus in Syria [33], which corresponds to our study where we found a high value for calculus.

In the study of H. Kalsbeek and W.H. van Palenstein Helderman for oral health problems in children in Palmyra at 13–15 years of age, it was found that although oral care had been received, only 27% of the individuals studied were free of oral health diseases, and 29% suffered from non-treating painful caries, 24% suffered from gum bleeding and 24% suffered from orthotics and cosmetic problems. These numbers correspond to our study where we found that only 14% of the sample were free of oral disease [34].

Compared to the previous studies results, we find a decline in oral health during the Syrian crisis and an increase in the number of teeth decayed and teeth lost due to dental necrosis.

The average DMFT value in our study was 3.36, where this value exceeds the DMFT average for the 12-years age group in 1988 in Syria that were estimated as 1.9 and also higher than the 1994 study that has been estimated as 2.5 and more than in 1998 which has been estimated as 2.3 [35].

In comparison to the global average of DMFT 2000 for the same age group, which is estimated as 2.4, we notice that the value in this study is higher than the global average [36].

The study of 2014 in Lattakia in Syria showed that the value of DMFT was estimated as 2.35 for the age group 12 years [37], which is less than our study’s value. It may be due to a number of factors. The most important one is that the capital city – Damascus – was affected by a large set of Syrian crisis factors and changes in the economic situation for the population [37].

Regionally, a study was conducted in Eastern Saudi Arabia in 2014 showed the value of DMFT 2.0 for the 10–12 age group, which is less than our study’s value [38].

Recent studies have shown a relationship between (SES) and oral health, where the results showed a significant association between educational level and oral health [29].

In a study conducted in Iraq during the 2007 war on 12-years-old school children, dental treatments were significantly more pronounced in children with parents with a higher educational level and in populations with higher socio-economic status [39]. This is also reflected in the Casanova study of school children aged 6–13 years in Mexico, where children with mothers that have a higher educational level tended to care and brush their teeth more than children with a lower mother educational level [18].

Moreover, it has been shown that the type of school and grade reached by the child can affect his/her oral health. Therefore, it appears that a child who has completed a lower educational level may have a lack in oral health knowledge more than a child with a higher educational level. In addition, the child’s grades significantly affect his/her oral health and the amount of caries he/she has [40]. This coordinates with our current study that verifies the significant relationship between the educational level and oral health among children.

Schools can provide the best rules and basic concepts about oral health care, which helps children at this stage to establish and develop oral health acquisition skills [38].

This research studied the oral health among children during the years of the Syrian crisis, and the prevalence of the decayed, missed, or filled teeth among the sample. Despite the limitations of this research, it can be considered as a reference point for any future compression between the oral health among Syrian sample and the oral health during the time of the crisis. Also, the results of this research showed an association between the oral health and the level of the Socio-economic status among the Syrian children, which can be useful to direct any future governmental or nongovernmental oral health plans or awareness campaign to focus on the most needed areas and children from low socio-economic status.

## Suggestions and recommendations

1. Emphasize on the importance of oral health care for children.
2. Cooperation with schools and institutions that deals with children to provide the best oral health awareness services.
3. Work on the application of fluorinated materials to protect the teeth from necrosis.
4. Adopting a preventive educational program and focusing on the importance of educational attainment for children.
5. Emphasize the role of the parents in education and dissemination the health awareness among their children.
6. Establish a specific formula for cooperation between the University of Damascus, the Ministry of Education (MOE) and the Ministry of Health (MOH) to raise awareness among children.
7. Conducting additional educational studies for the oral health and its relation to the educational attainment level for children.

## Limitation

This research is limited to a certain sample size and certain timeframe, and the study design of this research makes the generalization of the results inappropriate.

## Conclusion

There are high levels of dental caries among children, and higher rate of dental caries can be predicted among children from a lower socioeconomic status, and the Syrian crisis could have played an important role in decreasing the oral health by changing the socioeconomic status of the people and by make it harder to access for the public health services. However, this study is limited to this sample and can not conclude a causal relation between the socioeconomic status and oral health, and that would need more research with different methodology.

## Data Availability

All necessary data are presented within the manuscript. All other materials and data are available upon request. For any more details regarding the data of this research please contact the corresponded author – dr. MHD bahaa Aldin Alhaffar.

## Abbreviations

DMFT: Decayed, missed, filled tooth
SES: Socio-economic status
